# Quantitative proteomics of rat and human pancreatic beta cells

**DOI:** 10.1016/j.dib.2015.02.019

**Published:** 2015-03-10

**Authors:** B. Brackeva, G. Kramer, J.P.C. Vissers, G.A. Martens

**Affiliations:** a B-Probe, Diabetes Research Center, Vrije Universiteit Brussel (VUB), Belgium; b Department of Clinical Chemistry and Radio-immunology, Universitair Ziekenhuis Brussel, Brussels, Belgium; c Department of Medical Biochemistry, Academisch Medisch Centrum, Amsterdam, The Netherlands; d Waters Corporation, Wilmslow, United Kingdom

## Abstract

Data set description: This data set is composed by label-free alternate-scanning LC-MS/MS proteomics analysis human and Wistar rat pancreatic islet endocrine cells.

The mass spectrometry data of the human and rat pancreatic beta cells and the resulting proteome search output from ProteinLynx GlobalSERVER (PLGS) have been deposited to the ProteomeXchange Consortium [1] via the PRIDE partner repository with the dataset identifiers PXD001539 (human) and PXD001816 (rat). From these mass spectrometry data, ‘relative molar amount units’ between cell types and across species were calculated.

Biological relevance: These data provide a quantitative view on the unfractionated proteomes of human and rat beta and alpha cells. It is likely biased towards the proteins with higher molar abundance, relating to core functional pathways, but also includes several proteins with an islet-enriched expression. The quality of the cell preps is state-of-the-art, and the label-free quantitation is both precise and accurate, allowing detailed quantitative analysis.

## Specifications table

**Table:** 

Subject area	Biology
More specific subject area	Rat and human pancreatic beta cell proteomics
Type of data	Excel tables
How data was acquired	Label-free alternate-scanning LC-MS proteomics; Nanoscale LC separation of tryptic peptides was performed with
	a nanoACQUITY system (Waters Corporation), equipped with a
	Symmetry C18 5 mm, 2 cm 6180 mm precolumn and an Atlantis
	C18 3 mm, 25 cm 675 mm or a Atlantis C18 3 mm, 15 cm
	675 mm analytical reversed phase column (Waters Corporation).
Data format	Raw/Analyzed
Experimental factors	Proteomes of rat alpha and beta cells were studied immediately after cell isolation, without in vitro culture or treatment. Proteins were typically extracted within 4 h of animal killing. Human beta cells were prepared from islet-enriched pancreas fractions, cultured for 6–14 days after human organ procurement. Selected fractions cleared for research were then trypsin-dissociated into single cell suspensions and FACS-sorted according to cell size, granularity and zinc content to an average insulin-purity of 60%. Proteomes of these cells was studied immediately after cell isolation without additional treatment or further culture.
Experimental features	Proteomes were recorded using label-free LC-MS/MS. Accurate mass precursor and fragment ion LC-MS data were collected in data independent, alternate scanning (LC-MSE) mode of acquisition [2,3]. Biological interpretation of the “relative molar amount units” was done after geometric normalization to a network of stable reference proteins [4–8]
Data source location	Rat data: Waters Corp., Manchester, UK, Analyst: J.P.C. Vissers
	Human data: Department of Medical Biochemistry, Academisch Medisch Centrum, Amsterdam, the Netherlands, Analyst: G. Kramer
Data accessibility	Raw data uploaded to ProteomeXchange Consortium via the PRIDE partner repository with the dataset identifiers
	**PXD001539: human data**
	Project DOI: 10.6019/PXD001539
	Reviewer account details:
	Username: reviewer99564@ebi.ac.uk
	Password: pJzPiCIj
	**PXD001816: rat data**
	Project DOI: 10.6019/PXD001816
	Username: reviewer43213@ebi.ac.uk
	Password: y2B5bYLW

## Value of the data

• The methods for FACS-purification of rat alpha and beta cells, and FACS-enrichment of human islet endocrine cells were developed by our lab, and results in state-of-the-art endocrine purities. This results in minimization of exocrine contamination, which is a problem affecting other proteomics studies on these cell types
• Human and rat beta cells were analyzed using exactly the same methods of protein extraction, trypsinization, LC-MS/MS analysis and downstream data analysis by the same people (Vissers and Kramer). This makes it possible for the first time to make a detailed quantitative comparison of the core proteomes of this cell type in two species.
• We applied rigorous statistical criteria for peptide/protein identification and quantification, thereby reducing false positive identifications perhaps at the expense of claimed coverage.
• We showed that alternate-scanning LC-MS/MS generated highly reproducible protein quantifications (with overall technical plus biological imprecision<20% and high accuracies as judged by correct measurement of multi-enzyme stoichiometry [4]).
• A limitation is our depth of coverage: with an average of 400–500 protein identifications/quantifications per cell type, our analysis is likely biased to the core proteome and proteins with higher molar abundance.

## 1 Data, full experimental design, materials and methods

Description of the analyzed islet endocrine cell preparations:

(i) FACS-purified rat insulin-producing beta cells (90% insulin+, 3% glucagon+, 1% somatostatin+ and 2% pancreatic polypeptide+ cells); *n*=3 biological replicates, 3 technical replicate injections each:
(ii) FACS-enriched rat glucagon-producing alpha cells (2% insulin+, 94% glucagon+, 1% somatostatin+ and 2% pancreatic polypeptide+ cells); *n*=3 biological replicates, 3 technical replicates each. Rat alpha and beta cells were FACS-purified as described [9,10]. Cells were processed for proteomics immediately after cell isolation without additional culture or treatment.
(iii) FACS-enriched human beta cell preparations (*n*=4 different donors, 3 technical replicates each) and
(iv) human pancreatic exocrine/duct cells (*n*=1 biological replicate, 4 technical replicates). Human beta cells were FACS-purified from pancreatic islet-enriched fractions that were prepared for clinical islet transplantation in non-uremic type 1 diabetic patients. Sporadic cell preparations not suitable for islet transplantation were cleared for research use. These cell preparations were typically cultured for 6–14 days in HamF10 supplemented with 6 mM glucose, 2 mM glutamine, 0.5% BSA, penicillin/streptomycin, with refreshing of the medium and removal of dead cells every 3 days. Islet-enriched fractions were dissociated into single cell suspensions, and viable endocrine cells FACS-isolated according to cell size, granularity and zinc content, to an average insulin-positive purity of 60±6% (range: 53–68%). These cells were then immediately processed for proteomics, exactly as done for the rat cells, without further treatment or culture.

Protein extraction After isolation, cell preparations were washed and soluble protein was extracted with 0.5% (w/v) RapiGest detergent in 50 mM ammonium bicarbonate in the presence of Complete Protease Inhibitor Cocktail and bovine DNAse II solution, followed by centrifugation to remove cellular debris. One human beta cell pellet was subjected to an additional re-extraction in 1% (w/v) Rapigest to verify if this resulted in higher recovery of hard-to-extract/membrane-bound proteins (HP2010, Table 1). To remove protease inhibitors and most of reduced insulin molecules, protein extract was reduced with 100 mM dithiothreitol (DTT). Proteins were denatured by heating at 80 °C for 15 min, followed by 30 min at 60 °C after addition of 2.5 µl 100 mM DTT and another 30 min at ambient temperature in the dark after addition of 2.5 µl 200 mM iodoacetamide. Trypsinization was carried out overnight at 37 °C (1:25 w/w trypsin ratio) in final volume of 100 µl. Finally, RapiGest detergent was removed by acidifying digest to pH=2 with trifluoroacetic acid and incubation for 15 min at 37 °C.

LC-MS configuration Nanoscale LC separation of the tryptic peptides was performed with a NanoAcquity system (Waters Corporation). Samples were loaded on to a Symmetry C18 5 μm, 2 cm×180 μm trap column (Waters) at a flow rate of 5 μl/min prior to separation on a Bridged Ethyl Hybrid C18 1.7 μm, 25 cm×75 μm analytical reversed-phase column (Waters) by application of a 90 min gradient from 1% ACN and 0.1% formic acid to 40% ACN and 0.1% formic acid at a column flow rate of 0.25 μl/min. The column temperature was maintained at 35 °C. Analysis of the eluted tryptic peptides was performed using a Synapt G2 Q-TOF (quadrupole time-of-flight) mass spectrometer (Waters Corporation) equipped with a nanolockspray source (Waters Corporation) fitted with a pico-tip emitter (New Objective) operated at a capillary voltage of approximately 3 kV. For all measurements, the mass spectrometer was operated in v-mode with a typical resolution of at least 20,000 full width at half maximum. All analyses were performed in positive mode ESI. The time-of-flight analyzer of the mass spectrometer was externally calibrated with a NaI mixture from m/z 50 to 1990. The collision gas used was argon, maintained at a constant pressure of 2.0×10−3 mbar in the collision cell. The lock mass, [Glu1]-fFibrinopeptide B, was delivered from the auxiliary pump of the NanoAcquity system with a concentration of 100 fmol/μl at 0.5 μl/min to the reference sprayer of the nanolockspray source. The data were post-acquisition lock-mass corrected using the monoisotopic mass of the doubly charged precursor of [Glu1]-Fibrinopeptide B, delivered through the reference sprayer, which was sampled every 120 s. Accurate mass precursor and fragment ion LC-MS data were collected in data independent, alternate scanning (LC-MSE) mode of acquisition [2,3]

LC-MS data processing and protein identification Continuum LC-MS data were processed and searched using ProteinLynx GlobalSERVER v2.5 (Waters Corporation). Protein identifications were obtained by searching databases of Rattus norvegicus databases (v15.12, 7449 entries) and Homo Sapiens release (2011_11, 20,335 entries). Sequence information of Alcohol dehydrogenase Saccharomyces cerevisiae was added to the databases to afford the ability to normalize the data sets and to estimate amounts and concentration and that of known contaminant proteins (e.g. serum albumin Bos taurus and trypsin Sus scrofa). A decoy was generated on the fly with every database search experiment conducted to estimate the protein false positive rate of identification. Data independent scanning protein identifications were accepted when more than three fragment ions per peptide, seven fragment ions per protein and more than 2 peptides per protein were identified, in at least one technical replicate per sample. Protein quantitation was only reported when the protein was detected in at least 2 out of 3 technical replicate of at least one biological replicate. Typical search criteria used for protein identification included automatic peptide and fragment ion tolerance settings (approximately 10 and 25 ppm, respectively), 1 allowed missed cleavage, fixed carbamidomethyl-cysteine modification and variable methionine oxidation. Raw data were expressed as ‘relative molar amount units’ calculated by dividing the determined molar amount for a given protein by the summed determined amount for all identified proteins as this accounts for both technical and biological variations [3,4,11]. To enable reuse of the continuum LC-MS data by other proteome search engines, MS1 and reconstructed MSn mass spectrometric data generated by PLGS were exported in mzML format.

Geometric normalization between cell types and across species: to compare these relative molar amounts between cell types and across species, data were biologically interpreted after geometric normalization to a set of stable housekeeping proteins, as described in the associated 4 Pubmed-indexed studies thus far: analysis of the rat beta cell proteome [4], identification of doublecortin [5], protein phosphatase 1-inhibitor 1 [6] and ubiquitin thioesterase-L1 [8] as candidate real-time biomarkers for beta cell destruction (Table 2).

## Figures and Tables

**Table 1:** Overview of all technical replicate LC-MS/MS injections of the indicated independent biological isolates. First column indicated the sample name, second column listed the codes of individual technical replicates, as used in the supplementary raw MZml data files.

**Human pancreas number**	**Code tech. Replicate**		**% insulin+**	**% dead**	**Rapigest**
HP2010 #3121	20111012_s4b	human beta cells, donor 1	**68**	4	0.50%
	20111103_s4a				
	20111103_s4c				
HP2064 #1421	20111103_s5a	human beta cells, donor 2	**62**	3	0.50%
	20111103_s5b				
	20111103_s5c				
HP2785 #2721	20111103_s6a	human beta cells, donor 3	**58**	2	0.50%
	20111103_s6b				
	20111103_s6c				
HP2789 #1821	20111103_s7a	human beta cells, donor 4	**53**	4	0.50%
	20111103_s7b				
	20111103_s7c				
HP2703 #2	20111103_s8a	human duct/exocrine	2	24	0.50%
	20111103_s8b				
	20111103_s8c				
HP2010 #3121 1% Rapigest	20111103_s16a	re-extraction of HP2010	68	4	1.00%
	20111103_s16b	at 1% Rapigest			
	20111103_s16c	(data not further used)			

Rat beta cells	Code tech. Replicate		% insulin+	% glucagon	Rapigest

beta 001	011108_002	biological replicate 1	90	3	0.50%
	011108_003	(pool of 25 animals)		(average)	
	011108_004				
beta 002	011108_010	biological replicate 2			
	011108_011	(pool of 25 animals)			
	011108_012				
beta 003	011108_019	biological replicate 3			
	011108_020	(pool of 25 animals)			
	011108_021				

Rat alpha cells	Code tech. Replicate		% insulin+	% glucagon	Rapigest

alpha 001	011108_006	biological replicate 1	2	94	0.50%
	011108_007	(pool of 25 animals)		(average)	
	011108_008	co-isolated with beta 001			
alpha 002	011108_014	biological replicate 2			
	011108_015	(pool of 25 animals)			
	011108_016	co-isolated with beta 002			
alpha 003	011108_022	biological replicate 3			
	011108_023	(pool of 25 animals)			
	011108_024	co-isolated with beta 003			

**Table 2:** Overview of total proteins identified and quantified by LC-MS/MS in the various cell preparations, along criteria specified in Materials & Methods.

		**rat**		**human**	
		**beta**	**alpha**	**beta**	**exocrine**
number of proteins identified	*n*≥1	506	417	462	372
number of proteins quantified	*n*≥2	465	353	413	300
GEO-normalized molar amount	highest	3.61	8.22	8.95	14.74
	lowest	0.02	0.02	0.01	0.06
	dynamic range	226	548	746	246
biological replicates (*x*)		3	3	4	1
technical replicates (*n*)		3	3	3	3
